# Supplementary material for: Prognostic Role and Clinical Significance of Tumor-Infiltrating Lymphocyte (TIL) and Programmed Death Ligand 1 (PD-L1) Expression in Triple-Negative Breast Cancer (TNBC): A Systematic Review and Meta-Analysis Study
Source: Diagnostics (Basel). 2020 Sep 17;10(9):704. doi: 10.3390/diagnostics10090704 (PMC7554852; doi:10.3390/diagnostics10090704)
Supplement: Supplementary file 1 [file diagnostics-10-00704-s001.pdf]

Supplementary

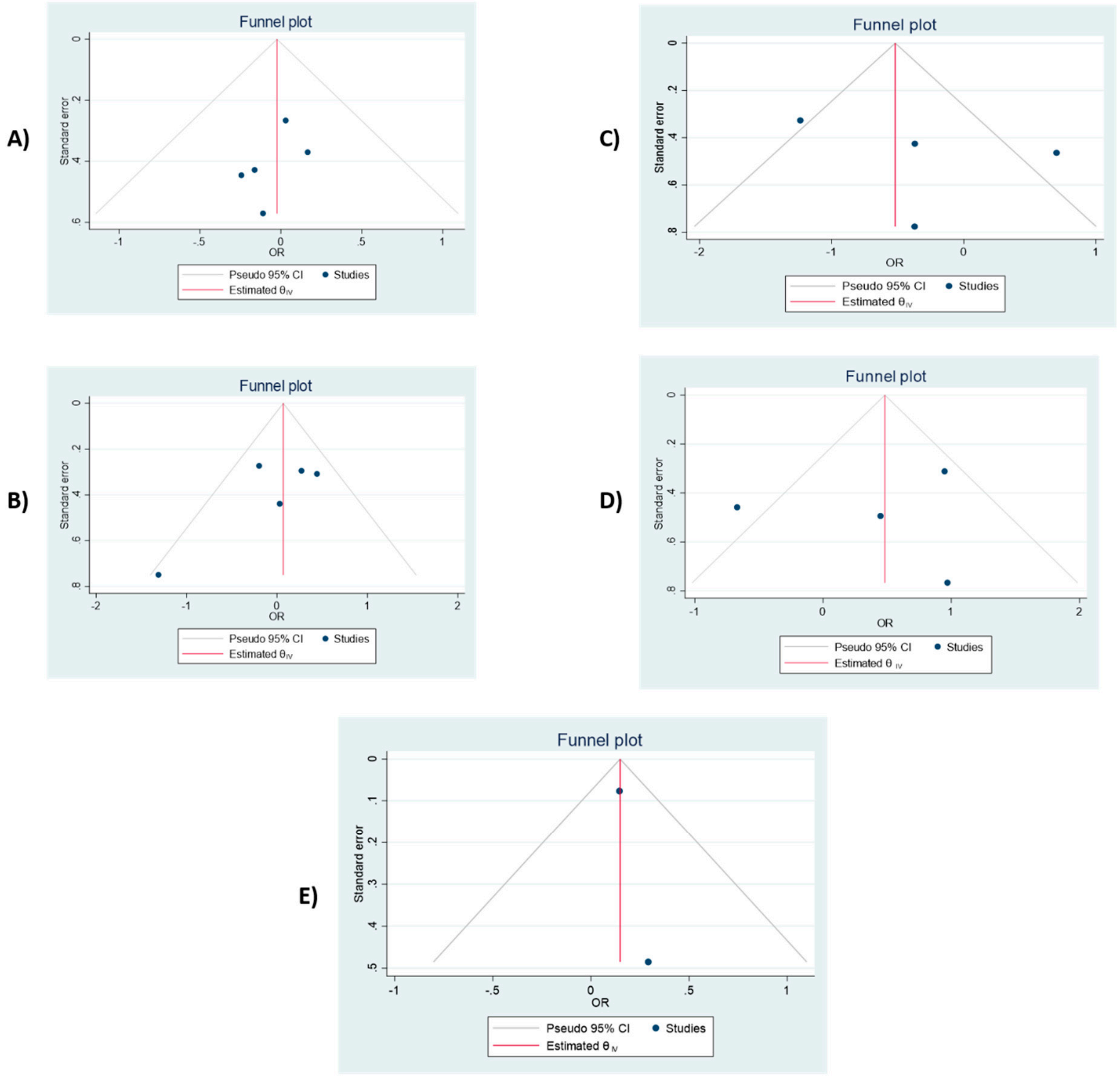

Figure S1. Funnel plots based on PD-L1 expression fortumor size(A), tumor stage (B),lymph node metastasis(C),tumor grade(D),Ki67 (E).

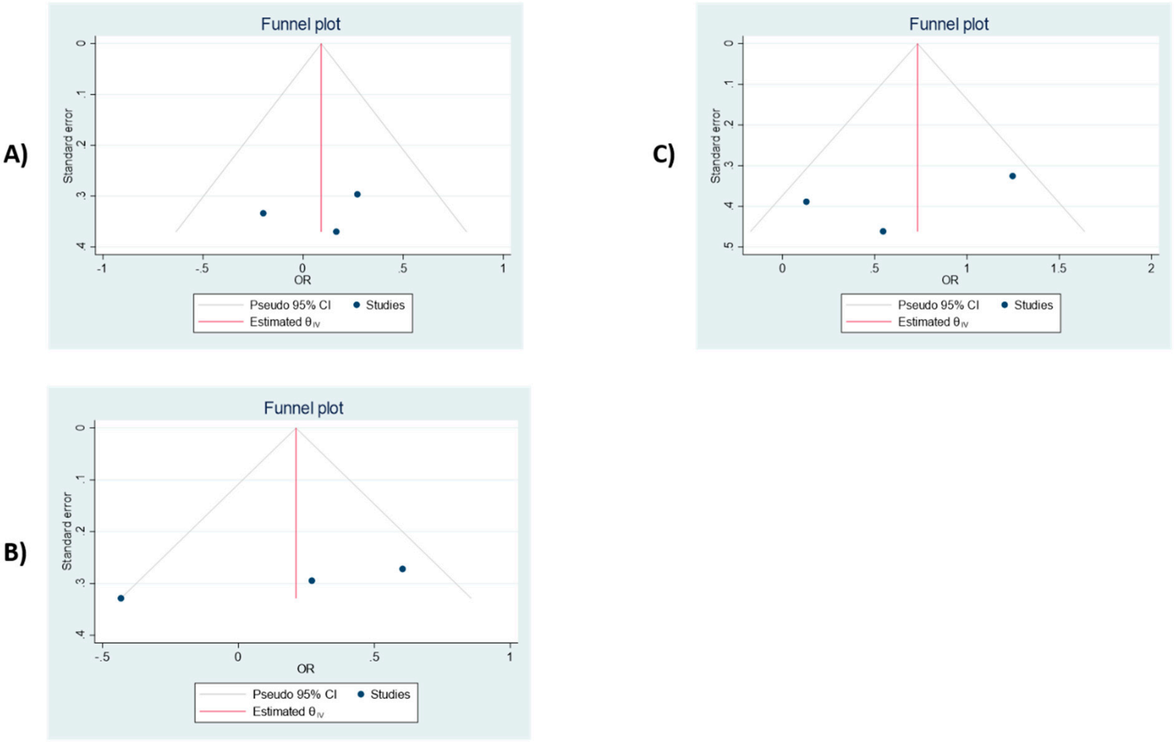

Figure S2. Funnel plots based on TILsfortumor size(A), tumor stage (B),lymph node metastasis(C).
